# Peer mentorship to build research capacity among members of the International Student Surgical Network (InciSioN): a proof of concept study

**DOI:** 10.1186/s12909-022-03482-9

**Published:** 2022-12-15

**Authors:** Ulrick Sidney Kanmounye, Soham Bandyopadhyay, Alejandro Munoz-Valencia, Halimah Khalil, Hamaiyal Sana, Nermin Badwi, Xiya Ma, Mamta Swaroop, Katayoun Madani

**Affiliations:** Research Department, Association of Future African Neurosurgeons, Kinshasa, Democratic Republic of Congo

**Keywords:** Capacity building, Global surgery, Medical student, Research

## Abstract

**Background:**

International Student Surgical Network (InciSioN) is the largest student and trainee global surgery interest group worldwide and its members have contributed significantly to global surgery research. The InciSioN Research Capacity-Building (IReCaB) program aimed to enhance the research skills and confidence of participants via a peer mentorship model.

**Methods:**

After an open call to members of InciSioN to enroll, participants’ knowledge of research methods and the process was evaluated through a test to assign mentor and mentee roles, with mentors being those who scored ≥ 20/25. Mentors then delivered a series of four webinars to help disseminate research methodology to mentees. Finally, mentees were tested on their knowledge of research and their satisfaction with the program was also evaluated.

**Results:**

Fifty-two participants, mostly from LMICs (*n* = 23/52, 44.2%) were enrolled, and 36 completed the program. There was a significant improvement in the proportion of questions answered correctly on the post-program test (*R* = 0.755, *p* < 0.001). Post-IReCaB test scores were greater than pre-IReCaB scores (*p* < 0.001). The difference in confidence after the course was also significant (*p* < 0.001). IReCaB participants successfully designed, implemented, and published an international cross-sectional study.

**Conclusion:**

This study showed improvements in participants’ understanding of theoretical components of scientific research. We offer a model for research capacity building that can be implemented, modeled, and further refined by similar organizations with academic research goals.

**Supplementary Information:**

The online version contains supplementary material available at 10.1186/s12909-022-03482-9.

## Background

Global health research quantifies the burden of disease and tests solutions that improve access to care. Study findings are most useful when they leverage the experience and expertise of local stakeholders [1]. Unfortunately, the current research capacity for conducting healthcare research in many low- and middle-income countries (LMICs), where the majority of the disease burden is recorded, remains limited and undermines the transformation of health systems into capable, self-sustaining ecosystems [2, 3]. The current distribution of health research capacity is disproportionately concentrated in high-income countries (HICs) [4, 5]. To address this imbalance, capacity-building through empowerment is essential among LMIC and HIC researchers.

Global surgery is a field at the intersection of global health and surgerical healthcare delivery systems encompassing clinical and non clinical specialties that shape the ecosystem of care for surgical patients [6]. Global surgery focuses on developing  equitable access to safe, timely, and affordable surgical healthcare worldwide and the field faces similar challenges as global health [7]. Global surgery trainees have contributed significantly to the design, data collection, data analysis, and dissemination of local and international landmark global surgery research projects, including the World Bank global surgery Indicators and International Research Collaboratives, such as, GlobalSurg and GlobalPaedSurg [8]. Although trainees are motivated to be involved in global surgery research, many if not most are limited by a lack of research skills or funding, especially those in low-resource settings [9–11]. Global surgery interest groups such as the International Student Surgical Network (InciSioN) provide a platform to share resources and knowledge amongst students, trainees, and early career healthcare professionals and surgeons worldwide [12–14]. InciSioN represents over 8000 students, trainees, and early career physicians around the world, who are passionate about equitable access to surgical healthcare worldwide.

### Research capacity context

In general, LMIC researchers and trainees are at a clear disadvantage in terms of research capacity-building due to a lack of resources [15]. In Africa for example, researchers lack funding (to conduct research, access published literature, and to attend and present at international conferences) and mentorship [9]. The research gap between LMICs and HICs is a hindrance in the scientific progress of global health research and research capacity-building in LMICs can help address this inequity [16]. On the other hand, HIC researchers often lack the insight and expertise to contextualize global surgery findings.

### Rationale and aim

This study aims to show that peer mentorship can help achieve respectable outcomes in research capacity-building programs. Each year, the InciSioN leadership evaluates its members' needs via a survey. During the 2020 needs assessment, InciSioN members expressed an interest in internal research capacity-building. As a result, InciSioN’s research team created an international student- and trainee-led online program to improve research capacity among participants via peer mentorship. The InciSioN Research Capacity Building (IReCaB) program participants were expected to learn and apply basic research methodology and biostatistics skills, learn about global surgery experiences from colleagues, and network with peers from different countries so they can form research collaborations in the future. These goals were set based on the organization’s experience participating in landmark global surgery research studies. The research skills and confidence in these skills were measured using tests and a Likert scale. IReCaB program participants were encouraged to propose research ideas and partner with colleagues from other countries.

## Methods

The work has been reported in line with the STrengthening the Reporting Of Cohort Studies in Surgery (STROCSS) and Checklist for Reporting Results of Internet E-Surveys (CHERRIES) criteria.

### Participants, definitions, and inclusion or exclusion criteria

Solicitation for enrollment in the IReCaB program was done through the InciSioN's WhatsApp channels and via email. Every InciSioN member taking the pre-IReCaB program test was included in the program. Participants were privy to their scores. The scores were used to set up peer mentorship groups between high performers, defined as a test score ≥ 20/25, and other performers. High performers assisted low performers in data analysis and academic writing.

The didactic component of IReCaB consisted of four webinars hosted on Zoom (Zoom Video Communications, California, USA) from April 25, 2020, to May 16, 2020, covering the basics of research design, data collection, data analysis, and research dissemination. Presenters were postdoctoral researchers from Africa, Europe, and Latin America with ≥ 10 peer-reviewed publications (USK, SB, AMV). The videos were recorded and then stored on InciSioN’s Google Drive (Google, California, USA) folder for asynchronous access. The webinars aimed to improve the median test score of enrollees by at least 7.5 (30%) points and the median confidence by at least 1.5 points by the end of the program [17].

The cohort was divided into seven groups of five to six participants 5–6 with high performers as group leads. The groups proposed studies and developed study protocols on global surgery themes. The themes covered access to service delivery, infrastructure, information management, and advocacy in general, thoracic, plastic, and transplant surgery as well as trauma and anesthesia. The protocols have been registered on open access registries and the research is ongoing. Another component of mentorship included one-onto-one exchanges between the program lead and low-performers to discuss the areas in which the low-performers showed little to no improvement. These conversations were hosted on WhatsApp and Zoom. 

### Data collection and validation

A pre- and post-program test, developed on Google Forms (Google, California, USA), was distributed to IReCaB program participants before and after the didactic component. The test was composed of 20 questions on research ethics, study design, data analysis (sampling, t-tests and regression), research tools (statistical packages and reference management software) and academic writing (references, reporting guidelines and post-publication dissemination). The test was developed to test basic research methodology and biostatistics concepts. Its face validity was ascertained by experienced faculty members with significant global surgery experience (> 20 research publications). The test was then piloted among non-IReCaB participants and feedback was obtained to improve the test’s operability and reliability. The test was scored on a scale of 25 points and each question was composed of an auxiliary question evaluating the participants’ level of confidence in their response. The level of confidence was evaluated using a 5-point Likert scale where lower scores represented lower confidence (Supplemental file 1, IReCaB test).

After the didactic component of IReCaB, all registrants were surveyed using a Google Form, to evaluate the satisfaction of the participants and garner their feedback (Supplemental file 2, satisfaction survey).

### Statistical tests

Spearman’s correlation test evaluated the relationship between overall pre- and post-IReCaB program test data. The responses were converted into a pre- and post-data format and analyzed using linear mixed modeling on SPSS v26 (IBM, New York, USA). Linear mixed modeling was used to identify the differences between and within subgroups. After excluding the data of dropouts, a dependent sample t-test was used with a compound effect to compute the covariance and the event (pre- or post-) as a fixed factor. The fixed effects were chosen because the data were collected at two-time points (pre- and post-IReCaB program). Parameter estimates (degrees of freedom, *p*-values, and the 95% confidence intervals) of fixed effects and estimates of covariance were generated. A *p*-value ≤ 0.05 was considered statistically significant.

## Results

**Fig. 1:**
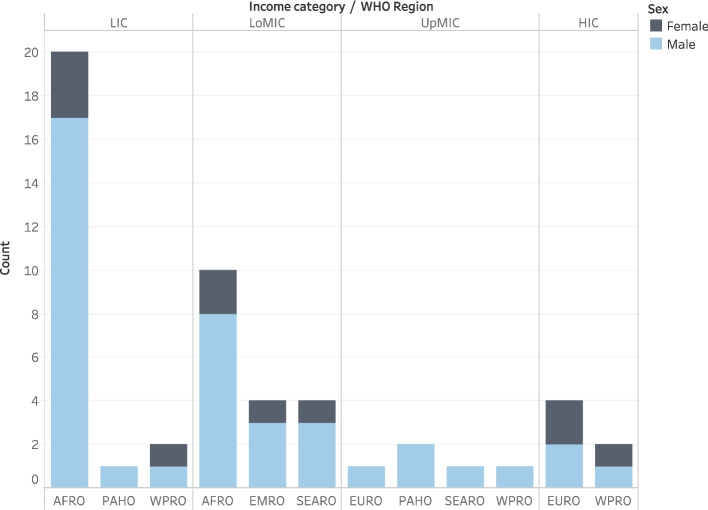
Distribution of the research capacity building enrollees by World Health Organization region and by country income category. AFRO—Africa, EURO—Europe, EMRO—Eastern Mediterranean, PAHO—Americas, SEARO—South East Asia, WPRO—Western Pacific

We enrolled 52 participants, the majority were male (78.8% *n* = 41/52), lived in low-income countries (44.2%, *n* = 23/52), and resided in Africa (57.7%, *n* = 30/52) (Fig. 1).

### Webinar logistics

Three of the four webinars were successfully conducted through Zoom as originally scheduled. One webinar was conducted using Zoom, at a different time, but was also recorded for asynchronous access. Presenters did not have issues joining webinars; however some participants did have unstable internet connections which at times impeded interactions with the presenter. All webinar recordings as well as slides were shared with participants through a Google Drive folder and the WhatsApp channel.

### Pre-/post-test

**Fig. 2:**
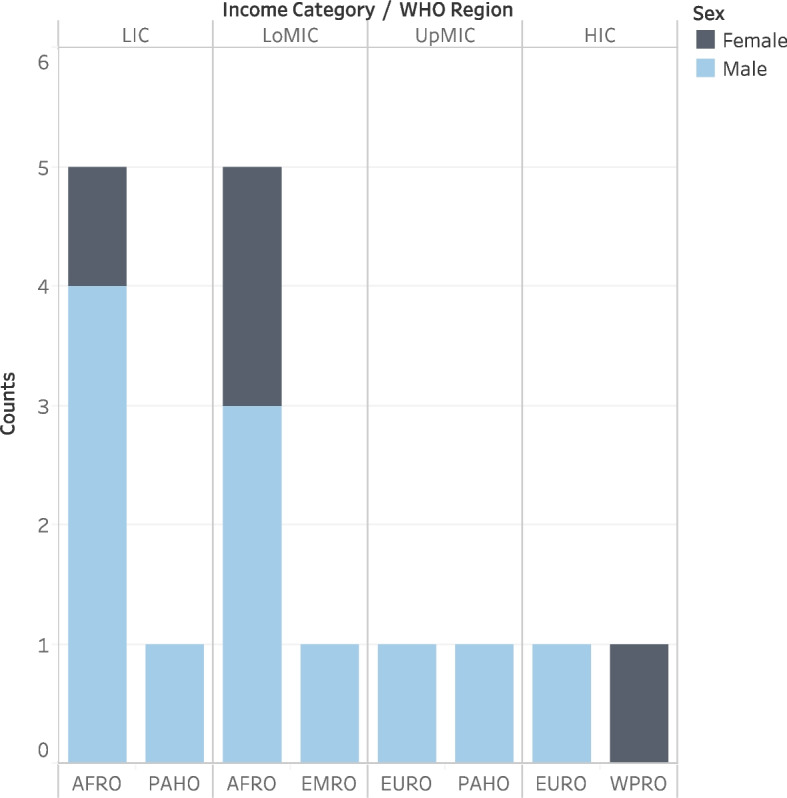
Distribution of the research capacity building dropouts by World Health Organization region and by country income category. AFRO—Africa, EURO—Europe, EMRO—Eastern Mediterranean, PAHO—Americas, SEARO—Southeast Asia, WPRO—Western Pacific

The median pre-IReCaB program test score was 12.0 (IQR = 7.5), the median level of confidence was 4.1 (IQR = 0.8), and the two measures correlated positively (*R* = 0.576, *p* < 0.001). Most participants could not define parametric data (94.2%, *n* = 49/52), identify free statistical packages (92.3%, *n* = 48/52) nor describe the calculation of an impact factor (88.5%, *n* = 46/52) (Table 1). Thirty-six (69.2%) participants attended all the lectures and 16 (30.8%) dropped out of the program. Most dropouts were male (75.0%, *n* = 12/16) and from Africa (62.5%, *n* = 10/16) (Fig. 2). The median pre-IReCaB program score of the dropouts was 6.0 (IQR = 5.0) and the median level of confidence was 4.0 (IQR = 1.4). The reasons for dropping out were lack of internet access (50.0%, *n* = 8/16), lack of study time (31.3%, *n* = 5/16) and language difficulties (18.8%, *n* = 3/16).

**Table 1:** Performance of the research capacity building enrollees in the pre- and post-IReCaB tests

Question theme	Proportion of wrong responses (Pre-IReCaB test) (%, (*N* = 52)	Proportion of wrong responses (Post-IReCaB test) (%, (*N* = 36))	Difference in the post- and pre-IReCaB proportion of wrong answers
1. Free statistical packages	92.3	77.8	-14.5
2. Regression	86.5	44.4	-42.1
3. Digital object identifier	11.5	5.6	-5.9
4. Referencing styles	69.2	41.7	-27.8
5. Reporting guidelines	61.5	19.4	-42.1
6. Sample size calculation	51.9	19.4	-32.5
7. Manuscript submission	63.5	52.8	-10.7
8. Hierarchy of evidence	44.2	13.8	-30.4
9. Research ethics	86.5	55.6	-30.9
10. Quantitative variables	73.1	22.2	-50.9
11. PRISMA flow diagram	86.5	2.8	-83.7
12. Impact factor	88.5	36.1	-52.4
13. Open access	3.8	2.8	-1.0
14. Parametric data	94.2	69.4	-24.8
15. ResearchGate	21.2	8.3	-12.9
16. Manuscript editing	38.5	22.2	-16.3
17. *P*-value	28.8	11.1	-17.7
18. Sensitivity	55.8	19.4	-36.4
19. Sampling	30.8	8.3	-22.2
20. Bias	67.3	36.1	-31.2

The median post-IReCaB program test score was 19.0 (IQR = 6.3) and the median level of confidence was 4.8 (IQR = 0.5). Similarly, to the pre-IReCaB program test, participants were unable to define parametric data (69.4%, *n* = 25/52) or identify free statistical software (77.8%, *n* = 28/52) (Table 1).

There were significant improvements in the proportion of questions answered correctly (*R* = 0.755, *p* < 0.001). The most important change in performance was noted on the questions about the PRISMA flow diagram (+ 83.7%), impact factor (+ 52.4%), sample size calculation (+ 42.1%), and regression analysis (+ 42.1%) (Table 1).

The post-IReCaB program test scores were higher than the pre-IReCaB program score (5.94, 95% CI = 4.17—7.72) for the participants completing the IReCaB program (Covariance = 11.04, SE = 4.59, df = 35, *p* < 0.001). Also, the difference in the level of confidence post- and pre-IReCaB program (0.71, 95% CI = 0.45—0.96) was statistically significant (Covariance = 3.11, SE = 1.27, df = 35, *p* < 0.001).

### From theory to practice

Multiple participants of the IReCaB program submitted and later presented their research outcomes to reputable conferences such as the Clinical Congress of the American College of Surgeons [18].

## Discussion

The study presented here is unique in that it used peer mentorship to develop research capacity and confidence among trainees dispersed across the globe. Additionally, the IReCaB research training program is unique to global surgery because medical students and trainees have been instrumental in the field’s progress. Traditionally, students have helped collect data and disseminate findings; however, they have expressed interest in other aspects of the research cycle: study design, data analysis, and project management. The IReCaB program showed that medical students and trainees interested in global surgery can handle these tasks if given the opportunity. Many of them developed successful studies and saw their projects to completion and presentation. 

The program saw greater participation from LMICs, mainly from African countries. After completing the IReCaB program, participants gained a better understanding of the theoretical and practical components of scientific research and were able to assert their opinions with more confidence. Although not meeting the set goals (≥ 7.5 test points and ≥ 1.5 confidence level), major improvements were observed in the median test score which increased from 12.0 to 19.0 (5.94, 95% CI = 4.17—7.72), and the confidence level increased from 4.1 to 4.8 (0.71, 95% CI = 0.45—0.96). The understanding of the PRISMA flow diagram, calculation of an impact factor and sample size as well as an understanding of regression analysis showed the most significant improvement.

The IReCaB program recruited members of InciSioN’s vast network and the program was designed to address the research needs of this population over four consecutive weeks. Previous studies found significant research capacity being built through rather abbreviated but structured programs. Namely, in 2008, a short program for research capacity building organized at Washington University in St. Louis demonstrated similar improvement in results in the participants' research strengths after the capacity-building intervention [19].

Research knowledge is a pivotal element of medical education, and through the study we found peer education to be a successful facilitator to making such knowledge available to medical students who otherwise are not privy to these resources. The study highlights the crucial role capacity-building initiatives play in nurturing research interest and knowledge by employing peer leadership.

Interest in global surgery is exponentially increasing and is particularly noticeable in research where a subfield called Academic Global Surgery (AGS) developed organically [20]. AGS is particularly popular among medical students and trainees [21]. AGS involves the application of research, education, and evidence-based advocacy toward surgical healthcare in regions with health inequities [22]. The IReCaB program experience may encourage public and private sectors to start investing in research capacity-building practices utilizing basic resources. Such strategies should follow a curriculum addressing the research needs of students regardless of their geographical and sociodemographic situations.

### Limitations and future directions

The study had several limitations. Participants were not enrolled in a systematic fashion; conversely, they responded to an open call within the InciSioN organization. Also, the assessments were conducted immediately before and after the IReCaB program. Therefore, it was not possible to evaluate any lasting impact on participants in terms of research projects, publications and other academic products. Other limitations restrict the interpretation and generalizability of results. Namely, the differences might be partially explained by unobserved confounders such as concurrent research training at a participant’s school. Further studies might use difference-in-differences analysis to evaluate the effects of a research capacity-building project. Whilst it is inherently difficult to assess the institutional impact of the IReCaB program and the resulting degree of contributions from participants to InciSioN research, this may be an avenue to explore in future evaluations. Although not gathered formally, most participants were French or native African speakers and therefore had language constraints.

Of the 52 participants, 16 (30.8%) dropped out of the program. Most of them were from Africa and had pre-IReCaB program test scores which were significantly lower than those of the other participants, 6/25 drop out cohort vs.12/25 non-drop out cohort.

Time zones were taken into consideration when designing the IReCaB program and the video lectures were made available via Google Docs (Google, California, USA). However, limited internet access meant those at high risk of dropping out could not attend the live lectures or watch the videos online. Another important challenge was language barriers. To adequately address difficulties surrounding the issue of language barriers, and variable time zones amongst participants, both reported as reasons for drop-out, the framework should be adapted to ensure inclusivity. Moving forward, similar capacity-building initiatives might benefit from providing supplementary written material in a variety of languages for participants to review in their own time. Likewise, the provision of additional resources in the form of written notes will allow participants to partake in the course materials as is desired despite unreliable internet connection. To encourage live attendance to webinars and maximize engagement from participants, where possible, sessions should occur in triplicate taking into consideration variable time zones. Overall, the characteristics of the dropouts and their reasons highlight the additional barriers faced by medical students in LMICs beyond inadequate or non-existent research infrastructure, lack of funding, and limited research exposure.

## Conclusion

The IReCaB program experience in developing research capacity among InciSioN members shows that peer mentorship can help achieve respectable results in research capacity-building. The authors found significant improvements in participant understanding of both theoretical and practical components of scientific research that can be achieved through a structured short-term program. This framework can be implemented, remodeled, and further refined by organizations with similar academic research goals. This model would be particularly relevant to groups whose members have diverse experiences as it leverages the strengths of individual members to improve the group.

## Supplementary Information

**Additional file 1.** IReCaB research quiz 1.

**Additional file 2.** IReCaB Satisfaction - Enrollee.

## Data Availability

The datasets generated and/or analyzed during the current study are available in the Open Science Framework repository, DOI: 10.17605/OSF.IO/9PBDV.

## Abbreviations

HIC: High-Income Country
IReCaB: InciSioN Research Capacity-Building program
InciSioN: International Student Surgical Network
LMIC: Low- and Middle-Income Country
