# Supplementary material for: Peer mentorship to build research capacity among members of the International Student Surgical Network (InciSioN): a proof of concept study
Source: BMC Med Educ. 2022 Dec 15;22:868. doi: 10.1186/s12909-022-03482-9 (PMC9753241; doi:10.1186/s12909-022-03482-9)
Supplement: Supplementary file 1 — Additional file 1. IReCaB research quiz 1. [file 12909_2022_3482_MOESM1_ESM.pdf]

IReCaB research quiz 1

*Required

1. Email *

2. Name *

Statistical packages

3. Which of the following are free statistical packages? * 1 point

Tick all that apply.

- [ ] R
- [ ] EpiData
- [ ] Zotero
- [ ] Epi Stats
- [ ] SPSS
- [ ] Stata

4. How confident are you with the answer you provided? *

Mark only one oval.

	1	2	3	4	5	
I am not sure	○	○	○	○	○	I am 100% certain

Regression

5. Which of the following are types of regression? * 1 point

Tick all that apply.

- [ ] Linear regression
- [ ] Logistic regression
- [ ] Poisson regression
- [ ] Cox Harzard regression

6. How confident are you with the answer you provided? *

Mark only one oval.

	1	2	3	4	5	
I am not sure	◯	◯	◯	◯	◯	I am 100% certain

Literature search

7. DOI stands for * 1 point

Mark only one oval.

- ◯ Data Optimization and Inference
- ◯ Data and Object Identification
- ◯ Descriptive Optimization Inference
- ◯ Digital Object Identifier

8. How confident are you with the answer you provided? *

Mark only one oval.

	1	2	3	4	5	
I am not sure	◯	◯	◯	◯	◯	I am 100% certain

References

9. Which of the following are reference styles? * 1 point

Tick all that apply.

- [ ] Harvard
- [ ] End note
- [ ] Vancouver
- [ ] AMA
- [ ] APA
- [ ] Zotero
- [ ] Mendeley

10. How confident are you with the answer you provided? *

Mark only one oval.

	1	2	3	4	5	
I am not sure	◯	◯	◯	◯	◯	I am 100% certain

Guidelines

11. The following are guidelines for reporting medical research except * 1 point

Mark only one oval.

- ◯ STROBE
- ◯ ICJME
- ◯ PRISMA
- ◯ CONSORT

12. How confident are you with the answer you provided? *

Mark only one oval.

	1	2	3	4	5	
I am not sure	⬭	⬭	⬭	⬭	⬭	I am 100% certain

Sample size

13. The sample size is determined by all of the following except * 1 point

Mark only one oval.

- ⬭ Power
- ⬭ Effect size
- ⬭ Population size
- ⬭ Confidence level
- ⬭ Mortality

14. How confident are you with the answer you provided? *

Mark only one oval.

	1	2	3	4	5	
I am not sure	⬭	⬭	⬭	⬭	⬭	I am 100% certain

Manuscript writing

15. When submitting an article you will need the following documents * 1 point

Tick all that apply.

- [ ] Cover letter
- [ ] Manuscript
- [ ] Transcripts
- [ ] Figures

16. How confident are you with the answer you provided? *

Mark only one oval.

	1	2	3	4	5	
I am not sure	⬭	⬭	⬭	⬭	⬭	I am 100% certain

Hierarchy of evidence

17. Classify the following in order of hierarchy of evidence (1 is the highest, 6 is the lowest) * 6 points

Mark only one oval per row.

	1	2	3	4	5	6
**Expert opinions**	⬭	⬭	⬭	⬭	⬭	⬭
**Cohort studies**	⬭	⬭	⬭	⬭	⬭	⬭
**Cross sectional studies**	⬭	⬭	⬭	⬭	⬭	⬭
**Case reports**	⬭	⬭	⬭	⬭	⬭	⬭
**Randomized controlled trials**	⬭	⬭	⬭	⬭	⬭	⬭
**Case-control studies**	⬭	⬭	⬭	⬭	⬭	⬭

18. How confident are you with the answer you provided? *

Mark only one oval.

	1	2	3	4	5	
I am not sure	◯	◯	◯	◯	◯	I am 100% certain

Ethics

19. Which of the following qualify as unethical research practices? * 1 point

Tick all that apply.

- [ ] Submit an article to multiple journals at the same time
- [ ] Modify the lighting or color on a pathology slide or microscopic image
- [ ] Award authorship to an acquaintance to thank them for their support and mentorship
- [ ] Use results from one of your previous studies without referencing them

20. How confident are you with the answer you provided? *

Mark only one oval.

	1	2	3	4	5	
I am not sure	◯	◯	◯	◯	◯	I am 100% certain

Types of variables

21. Which of the following are types of quantitative variables? * 1 point

Tick all that apply.

- [ ] Nominal
- [ ] Continuous
- [ ] Discrete
- [ ] Ordinal

22. How confident are you with the answer you provided? *

Mark only one oval.

	1	2	3	4	5	
I am not sure	( )	( )	( )	( )	( )	I am 100% certain

Types of research

23. This flow diagram is an output of a * 1 point

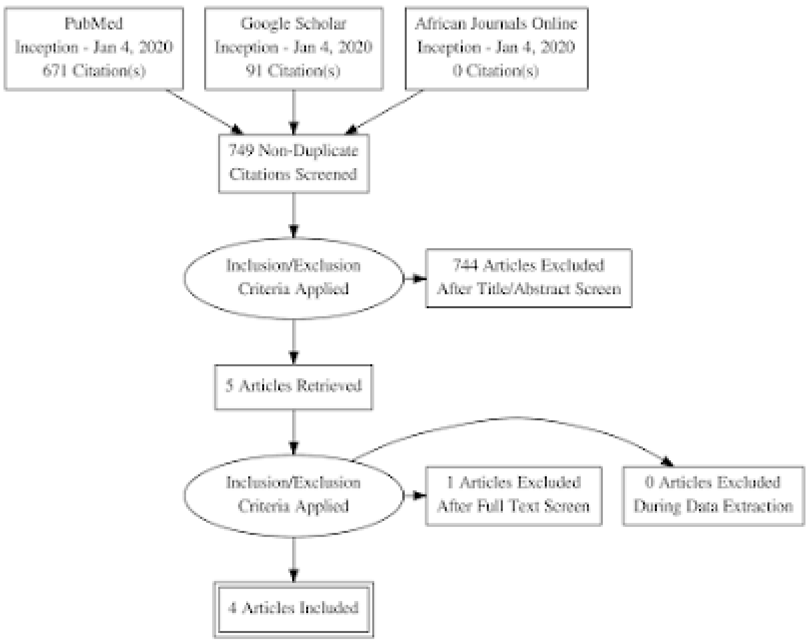

Fig. 1 PRISMA flow diagram of systematic review on neurosurgical patient perceptions in low- and middle-income countries outlining the number of initial non-duplicate citations screened, studies excluded, and the final 4 articles.

Mark only one oval.

- Case-control study
- Op-ed
- Systematic review
- Randomized controlled trial

24. How confident are you with the answer you provided? *

Mark only one oval.

	1	2	3	4	5	
I am not sure						I am 100% certain

Dissemination

25. The impact factor of journal is calculated from its * 1 point

Tick all that apply.

- [ ] Prestige
- [ ] Citations
- [ ] Number of articles published previously by the journal
- [ ] Scientific validity

26. How confident are you with the answer you provided? *

Mark only one oval.

	1	2	3	4	5	
I am not sure	◯	◯	◯	◯	◯	I am 100% certain

Dissemination

27. Open access is a mechanism by which research outputs are distributed online, free of cost or other access barriers. * 1 point

Mark only one oval.

◯ True

◯ False

28. How confident are you with the answer you provided? *

Mark only one oval.

	1	2	3	4	5	
I am not sure	◯	◯	◯	◯	◯	I am 100% certain

Data analysis

29. Parametric data has * 1 point

Tick all that apply.

- [ ] A normal (Gaussian) distribution
- [ ] The mean equal to the median
- [ ] A large sample
- [ ] Has 95% of the population within 2 standard deviations

30. How confident are you with the answer you provided? *

Mark only one oval.

	1	2	3	4	5	
I am not sure	⬭	⬭	⬭	⬭	⬭	I am 100% certain

Dissemination

31. ResearchGate is a social media platform for researchers * 1 point

Mark only one oval.

- ⬭ True
- ⬭ False

32. How confident are you with the answer you provided? *

Mark only one oval.

	1	2	3	4	5	
I am not sure	⬭	⬭	⬭	⬭	⬭	I am 100% certain

Manuscript writing

33. The following are sections of a manuscript * 1 point

Tick all that apply.

- [ ] Abstract
- [ ] Discussion
- [ ] Methods
- [ ] Introduction
- [ ] Results
- [ ] Conclusion

34. How confident are you with the answer you provided? *

Mark only one oval.

	1	2	3	4	5	
I am not sure	⬭	⬭	⬭	⬭	⬭	I am 100% certain

Data analysis

35. What is the usual p-value? * 1 point

Mark only one oval.

- ⬭ 1%
- ⬭ 10%
- ⬭ 5%
- ⬭ 50%

36. How confident are you with the answer you provided? *

Mark only one oval.

	1	2	3	4	5	
I am not sure	⬭	⬭	⬭	⬭	⬭	I am 100% certain

Data analysis

37. Sentitivity * 1 point

Mark only one oval.

- ⬭ measures the proportion of actual positives that are correctly identified as such
- ⬭ measures the proportion of actual negatives that are correctly identified as such
- ⬭ the probability that subjects with a positive screening test truly have the disease
- ⬭ the probability that subjects with a negative screening test truly don't have the disease

38. How confident are you with the answer you provided? *

Mark only one oval.

	1	2	3	4	5	
I am not sure	⬭	⬭	⬭	⬭	⬭	I am 100% certain

Research design

39. A set of individuals selected from a statistical population by a defined procedure is a * 1 point

Mark only one oval.

- Sample
- Target population

40. How confident are you with the answer you provided? *

Mark only one oval.

	1	2	3	4	5	
I am not sure						I am 100% certain

Research design

41. Which of the following are forms of bias * 1 point

Tick all that apply.

- [ ] Individuals being more likely to be selected for study than others
- [ ] Differences in the accuracy or completeness of participant recollections of past events
- [ ] Researcher subconsciously influences the experiment

42. How confident are you with the answer you provided? *

Mark only one oval.

	1	2	3	4	5	
I am not sure						I am 100% certain

This content is neither created nor endorsed by Google.

Google Forms
