# Supplementary material for: Peer mentorship to build research capacity among members of the International Student Surgical Network (InciSioN): a proof of concept study
Source: BMC Med Educ. 2022 Dec 15;22:868. doi: 10.1186/s12909-022-03482-9 (PMC9753241; doi:10.1186/s12909-022-03482-9)
Supplement: Supplementary file 2 — Additional file 2. IReCaB Satisfaction - Enrollee. [file 12909_2022_3482_MOESM2_ESM.pdf]

IReCaB Satisfaction - Enrollee

Thank you for participating in IReCaB. We will like to take a few minutes of your time to get your opinion on this project. We value your feedback.

This survey is anonymous and confidential.

*Required

1. How satisfied are you with the teaching sessions? *

Mark only one oval.

	1	2	3	4	5	
Unsatisfied	◯	◯	◯	◯	◯	Extremely Satisfied

2. How relevant are the teaching sessions? *

Mark only one oval.

	1	2	3	4	5	
Irrelevant	◯	◯	◯	◯	◯	Extremely relevant

3. How satisfied are you with the support you get from the IT members? *

Mark only one oval.

	1	2	3	4	5	
Unsatisfied	◯	◯	◯	◯	◯	Extremely Satisfied

4. How satisfied are you with your development? *

Mark only one oval.

	1	2	3	4	5	
Unsatisfied	⬭	⬭	⬭	⬭	⬭	Extremely Satisfied

5. Would you recommend IReCaB to a friend? *

Mark only one oval.

- ⬭ Yes
- ⬭ No
- ⬭ Maybe

6. How would you rate your learning experience? *

Mark only one oval.

	1	2	3	4	5	
Terrible	⬭	⬭	⬭	⬭	⬭	Amazing

7. In your own words, describe how you feel about IReCaB *

____

____

____

____

____

8. What has been your biggest challenge so far? *

9. How can we improve your experience? *

This content is neither created nor endorsed by Google.

Google Forms
